# A new modification method for graphite felt electrodes in a MV/4-HO-TEMPO flow battery†

**DOI:** 10.1039/c9ra10966h

**Published:** 2020-02-11

**Authors:** Xinyu Li, Chengde Huang

**Affiliations:** Department of Applied Chemistry, School of Chemical Engineering and Technology, Tianjin University Tianjin 300072 P. R. China cdhuang@tju.edu.cn

## Abstract

Using graphite felt as the support body, reduced graphene oxide (rGO) is grown on the surface of carbon fibers by the hydrothermal reduction method, and the modified graphite felt was used as an electrode material and studied in a methyl viologen (MV)/4-hydroxy-2,2,6,6-tetramethylpiperidin-1-oxyl (4-HO-TEMPO) redox flow battery. This paper aims to solve the insufficient adhesion of the dip-coating method by simple, effective and low-cost means and provides a possibility for large-scale production and application; the new modification method increases the reaction area and performance of the electrode, resulting in high current density and improved battery performance, and in the current density of 60 mA cm^−2^, the battery provides 97.39% theoretical capacity, which has practical significance for battery configurations.

## Abstract 2

Graphene felt electrodes modified with reduced graphene oxide (rGO) can greatly improve the performance of MV/ 4-HO-TEMPO flow battery.

## Graphical abstract

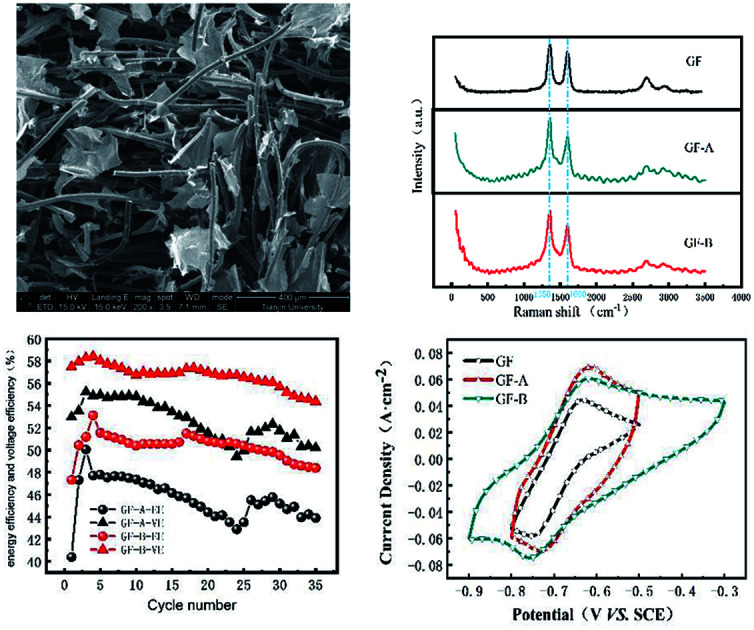

## 1. Introduction

Solar energy, wind energy, *etc.*, as green renewable energy sources are an important guarantee for the sustainable development of society, but due to their volatility and intermittent problems, they cannot be directly integrated into the traditional power grid. Thus, a new energy storage system—liquid flow is needed. A battery can provide amplitude modulation and frequency modulation to balance the output and ensure the operation of the power grid. Typically, a flow battery is composed of an electrode, an electrolyte, and an ion-exchange membrane. In particular, electrodes, the place where electrochemical reactions occur, are important for the electrochemical performance.^1^ Graphite felt (GF) is used in flow batteries due to its low cost, high conductivity, porosity, high stability, *etc.*, and GF can improve the power of battery; however, its application is limited due to its polarization problem. To improve the electrochemical activity of these carbon-based materials, considerable efforts have been made to modify the materials, such as functional group modification,^2–4^ optimization of the microstructure,^5,6^ increased active area,^7–9^ and catalyst loading.^10,11^

Graphene^12–16^ has attracted the attention of many researchers due to its high specific surface area, electrical conductivity and stability. In early 2011, Han *et al.*^17^ used graphene oxide (GO) nanosheets directly as electrodes for vanadium batteries, and the batteries exhibits less polarization; and higher conductivity and catalytic performance. In 2015, O. Di Blasi *et al.*^18^ used wet dip-coating methods to load GO powder on the surface of graphite felt, which was used as the electrode in a vanadium battery; and was found to have good catalytic activity. In 2016, Li *et al.*^19^ used vapor deposition to grow GO sheets on the surface of carbon fibers and formed a three-dimensional stable structure. This new modified electrode increased the VO^2+^/VO_2_^+^ redox reaction rate in the vanadium battery by three-fold, and the energy efficiency of the entire battery system increased by 11%. In 2017, González *et al.*^20^ used electrophoretic deposition to deposit GO sheets on the surface of graphite felt carbon fibers. The main performance indicator was that the battery had an energy efficiency of 95.8% at a current density of 25 mA cm^−2^. After the study of GO, rGO was also applied to the electrode as a catalyst. In 2017, Aaron *et al.*^21^ used a flow deposition method to transfer a rGO suspension into an electrolyte reservoir and circulated it through a vanadium battery to achieve a current density three times greater than the 80% voltage efficiency. In 2019, Pooria Moozarm Nia *et al.*^22^ grew rGO on the surface of carbon felt by a simple one-step electrodeposition process with GO, and used the product as a positive electrode electrocatalyst for vanadium redox flow batteries. The energy efficiency was significantly improved by 12% at a current density of 60 mA cm^−2^. The various application modes of graphene in an electrode have been mentioned above. The dip-coating method is simple in operation, practical and easy to use, but the adhesion depends on weak van der Waals forces and resulting poor durability. From the viewpoint of performance and stability, vapor deposition methods relying on chemical bonds are the most desirable; however, the complicated and cumbersome preparation process of vapor deposition hinders its large-scale production and application. Electrophoretic deposition and flow deposition are new method to solve these problems, and some progress has been made. In this paper, we adopt a new idea of three-dimensional graphene self-assembly, using graphite felt as the base frame; and a hydrothermal reduction method to grow rGO on the surface of carbon fibers. The surface of the carbon fibers is coated with rGO to form a rGO layer structure between the voids, which can form a three-dimensional stable structure similar to a graphene aerogel and enhance the structural stability. Experiments show that rGO not only increases the specific surface area and catalytic performance of the electrode, but also has a stable structure. It is a simple, practical and promising electrode modification method.

## 2. Materials and methods

### 2.1. Preparation of graphene/graphite felt composite electrode

First, the graphite felt sample was pretreated. Block graphite felt with a size of 1 × 1 × 1 cm^3^ was cut and ultrasonically cleaned in water for 30 min to remove the impurities on the surface of the graphite felt, and the debris was removed by cutting. Drying was used for sample A, and microwave treatment was used for sample B (power 300 W, microwave treatment for 3 min).

The two pre-treated graphite felt electrodes were simultaneously placed in a GO solution prepared by the modified Hummer's method^23,24^ (60 mL, 5 mol L^−1^), and then, 10 mL of ethylenediamine was pipetted with a pipette in the mixture, which was stirred and sonicated for 60 min. The mixture was placed in a 100 mL Teflon reactor at room temperature and heated to 180 °C for 12 h. Then, the temperature of the reactor was lowered to room temperature, and the graphene/graphite felt composite electrode was removed. The sample was repeatedly washed with deionized water; and then immersed in a 20% aqueous solution of ethanol for 8 h; the process was repeated 3 times, and then the sample was removed. The sample was frozen with liquid nitrogen and dried by a freeze dryer for 48 h to finally obtain a graphene/graphite felt composite electrode, which were designated GF-A and GF-B, respectively.

### 2.2. Structural and physical characterization

The morphology of the samples was studied by FE-SEM (S-4800, Hitachi Limited, Japan), with an acceleration voltage of 3.0 kV. Raman spectra were recorded; from 350 to 4000 cm^−1^; on a Thermo Fisher DXR Confocal Raman Microprobe (Thermo Electron Corporation, America) using a laser. X-ray diffraction (XRD) patterns were recorded on a Rigaku D/max-2500V/PC with Cu Kα radiation (*k* = 1.5406) at a scanning speed of 5° min^−1^. The atomic carbon and oxygen contents on the surface were determined by XPS analysis with a Thermo ESCALAB 250XI equipped with an Al Kα (1486.6 eV) X-ray source.

### 2.3. Electrochemical characterization

The cyclic voltammograms and electrochemical impedance spectra were obtained using a three-electrode cell on a PARSTAT-2273 electrochemical workstation (Princeton Applied Research, America) at room temperature. The cell consisted of samples of GF, GF-A or GF-B as the working electrode, a saturated calomel electrode as the reference electrode and platinum gauze as the counter electrode. Both working electrodes were square shaped with the same area (1.0 × 1.0 × 1.0 cm). The electrolyte was an 8 × 10^−3^ mol L^−1^ MV and 4-HO-TEMPO solution containing 1 mol L^−1^ NaCl as the supporting electrolyte. All of the tests were performed in an air atmosphere.

### 2.4. Single cell test

The performances of the cells were evaluated by various parameters of a single cell. The geometric area of the electrodes was 2.0 × 2.0 × 1.0 cm^3^ and a Selemion-AMV anion exchange membrane was applied in the experiment to change the chloride ion. The thickness of the membrane was 120 μm, the resistance was approximately 2.8 Ω cm^2^, and the ion mobility was more than 96%.

The main ingredient of the single cell was high-purity graphite, which improved the conductivity of the cell. The electrolytes were 0.1 mol L^−1^ MV and 0.1 mol L^−1^ 4-HO-TEMPO in a 1 mol L^−1^ NaCl solution in 45 mL containers, respectively. The range of test voltage was from 0.1 V to 1.7 V, and the flow speed was 90 mL min^−1^; the experiment was performed using by LANHE system (CT2001A, China).

## 3. Results and discussion

### 3.1. Morphology and characterization of the materials

**Fig. 1:**
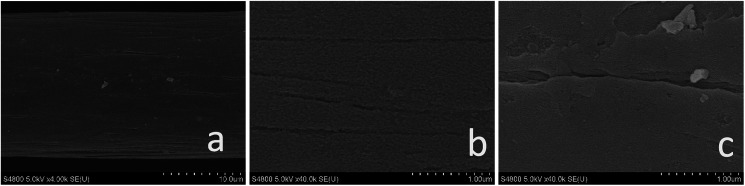
SEM images of (a) sample A, 4.0k magnification (b) sample A, 40.0k magnification (c) sample B, 40.0k magnification.

Fig. 1 shows the surface state of graphite felt after pretreatment. SEM images, which were enlarged 4.0k and 40.0k times, are shown in Fig. 1a and b, respectively, and are both sample A. The images show a smooth surface except for small gullies. In Fig. 1c, which shows sample B enlarged 40.0k times, it is clear that many fractures and large-area defects in two-dimensional plane are present in sample B.

**Fig. 2:**
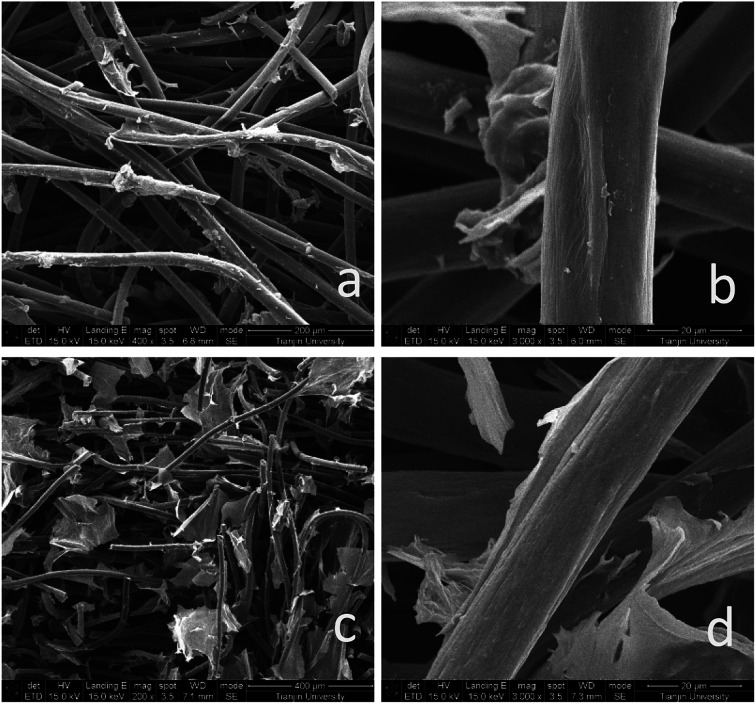
SEM images of: (a) GF-A, 400 times; (b) GF-A, 3000 times; (c) GF-B, 200 times; (d) GF-B, 3000 times.

Fig. 2 shows that significant changes occurred on the surface of the fibers, including the graphene/graphite felt composite electrodes GF-A (Fig. 2a and b) and GF-B (Fig. 2c and d). The surface of the carbon fibers were covered by rGO in Fig. 2b and d; however, denser rGO sheets were found for sample GF-B in Fig. 2c than in compared to the Fig. 2a. These sheets not only covered the surface of the fibers, but also acted as a connector in the gap between the fibers. This indicates that the microwave-treated carbon fibers contribute to the surface loading of rGO. In previous reports, microwave treatment has been shown to increase the content of –OH groups on graphite felt fibers and the roughness of graphite felt surfaces,^25^ while changes in surface morphology are generally considered to be defects from the formation of two-dimensional plane fractures. A large number of exposed two-dimensional structures may be more conducive to rGO self-assembly.

**Fig. 3:**
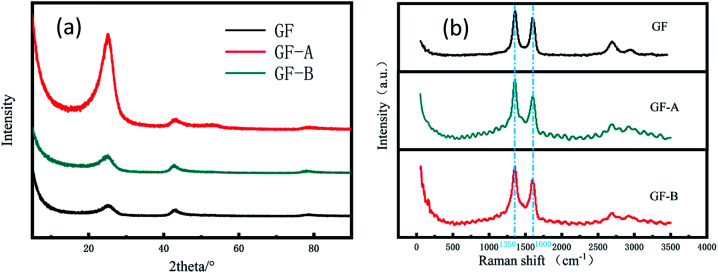
(a) XRD patterns of GF, GF-A and GF-B electrodes; (b) Raman spectra of GF, GF-A and GF-B electrodes.

In addition, Fig. 3a shows the XRD patterns of the samples. The sharp and intense peak positioned at 26° can be indexed to the (002) plane of graphite, and the peak of 43° is for the (100) plane. The patterns shown that the two peaks of the GF electrode have similar intensities, while the peaks of the GF-A and GF-B electrodes at 2*θ* = 26° are stronger than those at 43°, which may be due to a large amount of rGO being incorporated into the modified electrode.^26^

To further understand the changes in the surface morphology of the modified electrodes, a laser micro-Raman spectrometer was used for the characterization. It is well known that the Raman spectrum of carbon materials is mainly represented by a D peak at approximately 1350 cm^−1^ and a G peak at approximately 1590 cm^−1^. The G peak caused by the sp^2^ hybrid vibration of graphene is the main characteristic peak, which can effectively reflect the number of graphene layers.^27^Fig. 3b shows that the GF-A and GF-B samples have no significant shift in the G peak position compared to the untreated graphite felt after the addition of graphene, but the peak intensity increased, which may be caused by the incorporation of graphene sheets. The D peak is the disorder vibrational peak of graphene that characterizes the structural defects and edges of a carbon material. The peak position is generally 1350 cm^−1^, which can be compared with the D peak position of 1360 cm^−1^ for GF (Table 1). The peak position significantly shifts to the left, and the D peak shift is presumed to be caused by the incorporation of rGO. In general, the D peak of graphene is not obvious, which leads to a low peak value.^28^ A comparison shows the peak intensity of the GF-B sample is obviously weaker than that of the GF-A sample. Combined with Fig. 2c, it can be considered that this is because the content of rGO in GF-B is high after the material is wrapped with carbon fibers. The defects and edges of the carbon fibers in the original graphite felt are masked.

**Table 1:** Comparison of peak position and peak intensity in the Raman spectra of the three electrodes

Samples	D peak position	G peak position	D peak intensity	G peak intensity	*I*_D_/*I*_G_
GF	1360	1600	25.67	21.80	1.177
GF-A	1350	1600	38.52	30.93	1.246
GF-B	1350	1600	43.86	29.57	1.483

Although oxygen-containing functional groups can improve the hydrophilicity of the carbon fibers surface and become active sites for the redox reaction of the active material, a high content of the oxygen-containing functional groups will reduce the conductance of the carbon electrode material by increasing the ohmic resistance and charge transfer resistance of the carbon electrode, which leads to a decrease in the electrochemical performance.^29^

**Fig. 4:**
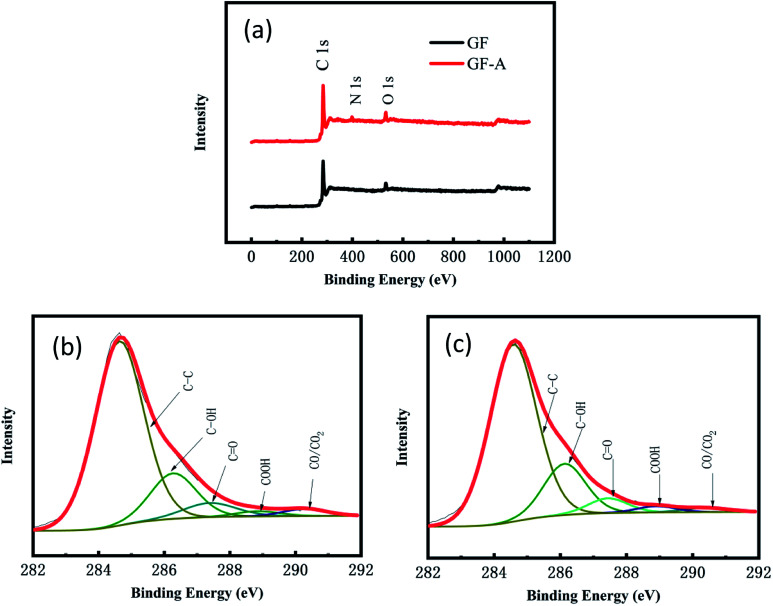
(a) XPS full spectra of the GF electrode and GF-A electrode; (b) schematic diagram of the C 1s deconvoluted peaks of the GF electrode; (c) schematic diagram of the C 1s deconvoluted peaks of the GF-A electrode.

Fig. 4a shows the full XPS spectra for the GF electrode and GF-A electrode. The GF electrode is mainly composed of carbon and oxygen, and the GF-A electrode is composed of three of carbon, oxygen and nitrogen (Table S2† for details). Comparing the GF and GF-A electrodes shows that the GF electrode has the highest oxygen content of 7.9%; however, in general, an ideal carbon electrode has an oxygen content in the range of 4 to 5%.^29^ Although the GF-A electrode is loaded with rGO and nitrogen is introduced to enhance the catalytic performance, the oxygen content only slightly decreases to 7.2%, indicating that the oxygen-containing functional groups may not be the main factor affecting the catalytic performance of the GF-A electrode.

Fig. 4b and c show a schematic diagram of the C 1s deconvoluted fitting peaks for the GF electrode and GF-A electrode. Five functional groups can be fit to the C 1s peaks: CC (284.6 eV), C–OH (286.1–286.3 eV), C=O (287.3–287.6 eV), –COOH (288.4–288.9 eV), and CO/CO_2_ (290.4–290.8 eV). The detailed functional group compositions of the samples are presented in Table S3 of the ESI.† These oxygen-containing functional groups will provide effective reaction sites for redox reactions.

### 3.2. Electrochemical performance

**Fig. 5:**
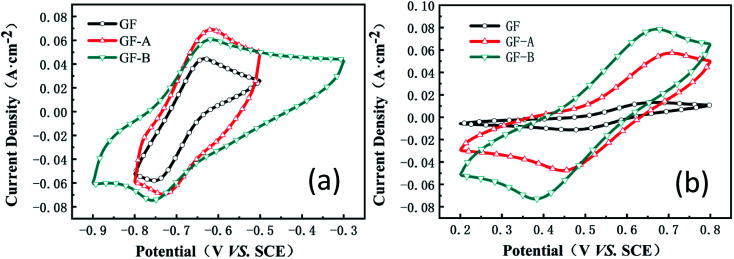
(a) CV curves of the GF, GF-A, and GF-B electrodes in MV solution; (b) CV curves of the GF, GF-A, and GF-B electrodes in 4-HO-TEMPO solution.

Fig. 5 shows the cyclic voltammetry (CV) curves for the GF, GF-A, and GF-B electrodes obtained at a scan rate of 50 mV s^−1^ (the values of the peak current, peak potential and peak potential difference are listed in Table S1, ESI†). Although the Δ*E* values of the anodic process for GF, GF-A and GF-B are almost the same, the peak current increases significantly; for example, the oxidation peak current increases from 0.04 A (GF) to 0.068 A (GF-A).

Compared to the higher reduction peak current of the GF-B electrode, the Δ*E* value of GF-B is obviously too large, and the smallest Δ*E* value among GF, GF-A, and GF-B indicates the considerably improved catalytic activity of the GF-A electrode. Similarly, for the cathodic process of GF, GF-A and GF-B, the peak current of all modified electrodes obviously improves. For example, the oxidation peak current increases from 0.013 A (GF) to 0.057 A (GF-A) and 0.078 A (GF-B). Microwave treatment can increase the number of –OH groups on the graphite felt carbon fibers, and according to Fig. 2a and c, the density of the rGO sheets on the modified electrode is different. The loading of rGO and the hydrophilicity both affect the catalytic activity of the GF-B electrode in the 4-HO-TEMPO solution. As the content of rGO increases, the active substance has more sites for the reaction, and the true specific surface area of the electrode increases, resulting in a large increase in current density (true specific surface area estimation in Fig. S1†). However, as the current density increases, the polarization also increases, according tothe Δ*E* value, which shows that the reversibility of the reaction on the electrode is affected and reduces the battery cycling.

**Fig. 6:**
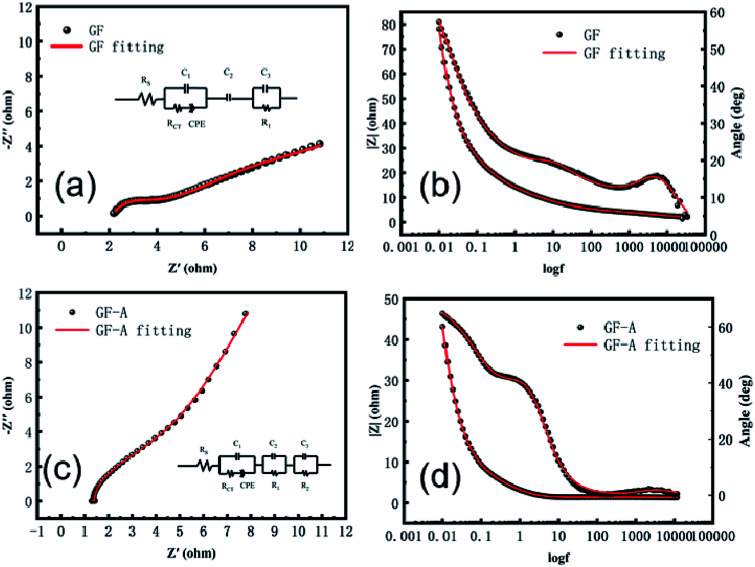
Electrochemical impedance spectroscopy of GF and GF-A electrodes; (a) Nyquist plot of the GF electrode; (b) Bode plot of the GF electrode; (c) Nyquist plot of the GF-A electrode; (d) Bode plot of the GF-A electrode.

To further study the electrochemical behaviour of the redox couple on graphite felt samples, electrochemical impedance spectroscopy of the GF electrode and GF-A electrode was measured at the open circuit potential in 8 × 10^−3^ mol L^−1^ 4-HO-TEMPO + 1.0 mol L^−1^ NaCl electrolyte. The related Nyquist and Bode plots are shown in Fig. 6. In the Bode diagram, the combination of the peaks and valleys of the phase angle is a time constant. It can be seen that there are two time-constants in Fig. 6b, which are due to the differences in the catalytic rate and mechanism of the 4-HO-TEMPO redox reaction on the electrode surface. It is likely that the oxygen-containing functional groups on the surface of the carbon fibers adsorb the 4-HO-TEMPO active molecule, so in the equivalent circuit, *C*_3_ and *R*_1_ represent the adsorption of 4-HO-TEMPO active molecules by oxygen-containing functional groups on the surface of the carbon fibers and the charge transfer resistance of the reaction, respectively, and *C*_2_ represents the adsorption of 4-HO-TEMPO active molecules by other oxygen-containing functional groups on the surface of the carbon fibers. Based on the equivalent circuit proposed by Shen *et al.*^30^ combined with the actual conditions in this experiment, the equivalent circuit (illustration in Fig. 6a) is simplified. In the equivalent circuit, *R*_s_ represents the ohmic resistance of the electrolyte and the electrode, *R*_ct_ is the charge transfer resistance of the redox reaction on the carbon fibers in the graphite felt, and CPE is a constant phase angle reflecting the diffusion capacitance generated by the reactive ion diffusion process.^30–32^ It can be seen from the figure that the fitted values agree well with the experimental values.

The GF-A electrode contains two carbon materials: carbon fiber in graphite felt and rGO sheets, which have different catalytic rates and reaction mechanisms for 4-HO-TEMPO redox reactions. Therefore, for the GF-A electrode, the equivalent circuit should contain three time-constants (Fig. 6d). Based on the equivalent circuit proposed by Shen *et al.*^30^ and the actual conditions of this experiment, the equivalent circuit (illustration in Fig. 6c) is simplified. In the equivalent circuit, *R*_s_ represents the ohmic resistance of the electrolyte and the electrode, *R*_ct_ is the charge transfer resistance of the redox reaction on the carbon fibers in the graphite felt, and CPE is a constant phase angle reflecting the diffusion capacitance generated by the reactive ion diffusion process.^30–32^*R*_2_ is the charge transfer resistance of the reaction for the redox reaction on rGO sheets, and *C*_3_ is the adsorption of 4-HO-TEMPO active molecules by the graphene sheets. *C*_2_ and *R*_1_ represent the adsorption of 4-HO-TEMPO active molecules by oxygen-containing functional groups on the surface of the carbon fibers and the charge transfer resistance of the reaction. It can be seen from the figure that the fitted value agrees well with the experimental value.

The beginning of the impedance diagram is a semicircle in the high-frequency region; the value at the intersection of the curve and the real axis is the ohmic impedance in solution, including the ohmic impedance of the electrode surface to the Luggin capillary and the impedance of the solution between the carbon fibers in the graphite felt electrode. Tables 2 and 3 show that the ohmic resistance of the GF-A electrode is close to one-half that of the GF electrode. The *R*_1_ values of the GF electrode and the GF-A electrode are similar, and the value of *R*_2_ is much smaller than *R*_1_. The rGO sheet has a small charge transfer resistance, greatly enhancing the electron transfer ability, which may be the reason for the catalytic enhancement of GF-A.

**Table 2:** Relevant electrochemical parameters obtained by fitting the Nyquist and Bode plots for GF

Sample	*R*_s_	*R*_ct_	CPE		*C*_2_	*R*_1_	*C*_3_
			Yo (S/sn)	*N* (0 < *n* < 1)			
GF	2.2	8.1 × 10^−1^	5.5 × 10^−2^	2.9 × 10^−1^	3.3 × 10^−1^	7.8 × 10^−1^	2.4 × 10^−2^

**Table 3:** Relevant electrochemical parameters obtained by fitting the Nyquist and Bode plots for GF-A

Sample	*R*_s_	*C*_1_	*R*_ct_	CPE		*R*_1_	*C*_2_	*R*_2_	*C*_3_
				Yo (S/sn)	*N* (0 < *n* < 1)				
GF-A	1.3	9.6 × 10^−2^	5.9	1.5 × 10^−1^	7.4 × 10^−1^	9.3 × 10^−1^	1.5 × 10^−1^	9.9 × 10^−2^	6.7 × 10^−4^

### 3.3. Single-battery performance

To further investigate this phenomenon, graphene/graphite felt composite electrodes were studied in a single-flow battery. Charge–discharge studies were carried out between 0.1 and 1.7 V. The experiment shows a decrease in the voltage loss and an increase in capacity with an increasing flow speed, according to Fig. S2 (ESI†), because the enhanced mass transfer reduces the concentration polarization on the electrode surface; as a result, a 90 mL min^−1^ flow speed was used.

**Fig. 7:**
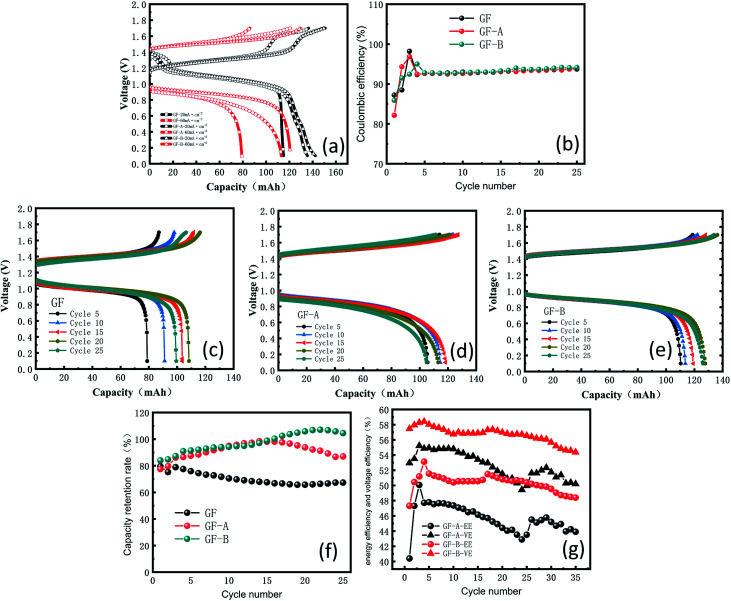
(a) Charge and discharge curves at 20 and 60 mA cm^−2^; (b) coulombic efficiency of GF and GF-A electrodes; (c) charge and discharge curves of the GF electrode at 60 mA cm^−2^; (d) charge and discharge curves of the GF-A electrode at 60 mA cm^−2^; (e) charge and discharge curves of the GF-B electrode at 60 mA cm^−2^; (f) capacity retention rate of GF, GF-A, and GF-B electrodes; (g) energy efficiency and voltage efficiency of GF-A and GF-B.

The main purpose of the electrode modification is to improve the power density, especially in the process of heavy current charging and discharging, so two distinct contrasting current densities, 20 and 60 mA cm^−2^, were selected to test the electrodes. Fig. 7a shows an obvious increase in capacity from GF to GF-A regardless of charging or discharging. The discharge capacity retention rate of the GF-A electrode reached 93.7% (Table S4, ESI†).

When 60 mA cm^−2^ was selected as the current density for multiple charge and discharge cycles, Fig. 7b shows the change in the coulombic efficiency (CE) for 35 cycles. The CE begins by sharply increasing, then decreases and eventually stabilizes at approximately 94%; the reason for the low CE at the beginning may be that the electrode is not completely wet or the electrode surface is not activated.^33,34^Fig. 7c–e show the changes in the capacity and voltage recorded every 5 cycles for GF, GF-A and GF-B. The figures show that GF-A and GF-B has more capacity during charge and discharge cycles than GF. Fig. 7f is the capacity retention rate (ratio of the actual capacity to the theoretical capacity) of the three electrodes in battery cycles. It can be seen from Fig. 7f that the maximum value for the GF electrode capacity is 95.99 mA h (79.6%), the minimum value is 79.3 mA h (65.75%), and the average capacity is 88.2 mA h (70.31%) in the discharge capacity of 25 cycles, and for the test of the GF-A electrode, the maximum capacity is 118.7 mA h (98.42%), the minimum value is 93.5 mA h (77.52%), and the average capacity is 108.8 mA h (90.21%) in the discharge capacity of 25 cycles. For GF-B, the values are 120.6 mA h (>99.99%), 101.18 mA h (83.90%) and 117.45 mA h (97.39%) in the discharge capacity of 25 cycles, respectively, which indicates the capacity retention rate of the modified battery at the same current density largely increases, and the values are 19.9% and 27.08% compared with the experimental results of Liu *et al.*^35^ For a current density of 60 mA cm^−2^ with 0.5 mol L^−1^ electrolyte solution, the battery provides 71.5% of the theoretical capacity (9.58 A h L^−1^ in 13.4 A h L^−1^). In the 0.1 mol L^−1^ electrolyte solution, the battery provides 57% of the theoretical capacity (1.54 of 2.68 A h L^−1^). The energy efficiency (EE) and voltage efficiency (VE) of the GF-A and GF-B electrodes were determined and compared (Fig. 7g), and GF-B was found to have higher EE and VE values, indicating better performance. It can be seen from the figure that VE and EE decrease by less than 2% after 35 cycles at a high flow rate of 90 mL min^−1^, indicating that the rGO adhesion on the surface of the carbon fiber is strong. The electrode after the cycles was selected for the SEM test (Fig. S3†). There are still many rGO sheets on the surface of the carbon fibers after 35 cycles.

## 4. Conclusions

In summary, graphene/graphite felt composite electrodes were successfully fabricated *via* a simple hydrothermal reduction method and used as an electrode in the MV/4-HO-TEMPO flow battery. Compared with previously reported graphene-based catalysts that were modified *via* the dip-coating and chemical vapor deposition methods, this unique method provides more active sites for MV/4-HO-TEMPO redox reactions by increasing the reaction area and costs less than the chemical vapor deposition method. Additionally, this method enhances adhesion between the graphene and the graphite felt electrodes. Removing the major disadvantage associated with the dip-coating method, in which the attachment between the material and the carbon fiber surfaces relies on weak van der Waals forces, this new method prevents destruction or disruption of the attachment by the flowing electrolyte, which can cause severe durability problems. Moreover, a significant improvement in current density in the CV test is obtained, and the battery releases more capacity when discharging at high current in the charge and discharge experiments. We believe this will be a new idea for modifying electrodes, which is of great significance for the cost and durability of the electrode.

## Conflicts of interest

The authors declare no competing financial interest.

## Supplementary Material

RA-010-C9RA10966H-s001
