# Supplementary material for: A new modification method for graphite felt electrodes in a MV/4-HO-TEMPO flow battery
Source: RSC Adv. 2020 Feb 11;10(11):6333–41. doi: 10.1039/c9ra10966h (PMC9049689; doi:10.1039/c9ra10966h)
Supplement: RA-010-C9RA10966H-s001 [file RA-010-C9RA10966H-s001.pdf]

Electronic Supplementary Material (ESI) for RSC Advances.
This journal is © The Royal Society of Chemistry 2020

Supporting Information for

A new modification method for graphite felt electrodes in a MV/ 4-HO-TEMPO flow battery

Xinyu Li, Chengde Huang*

Department of Applied Chemistry, School of Chemical Engineering and Technology, Tianjin University, Tianjin, 300072, PR China

E-mail: cdhuang@tju.edu.cn

Results and Discussion Section

(a)

Anode	I_{pa}	$-I_{pc}$	E_{pa}	E_{pc}	$I_{pa}/-I_{pc}$	△E
GF	0.04446	0.05803	-0.63399	-0.7549	0.7661	0.1209
GF-A	0.06896	-0.06941	-0.61557	-0.73248	0.9935	0.1169
GF-B	0.06063	-0.07465	-0.61682	-0.75855	0.8121	0.1417

(b)

Cathode	I_{pa}	$-I_{pc}$	E_{pa}	E_{pc}	$I_{pa}/-I_{pc}$	△E
GF	0.0132	-0.01119	0.67564	0.47089	1.1796	0.2048
GF-A	0.05728	-0.04759	0.7082	0.44555	1.1945	0.2627
GF-B	0.07854	-0.07295	0.67376	0.38149	1.0766	0.2922

Table S1 (a) The values of the peak current, peak potential and peak potential difference for anode (b) The values of the peak current, peak potential and peak potential difference for cathode

True specific surface area estimation

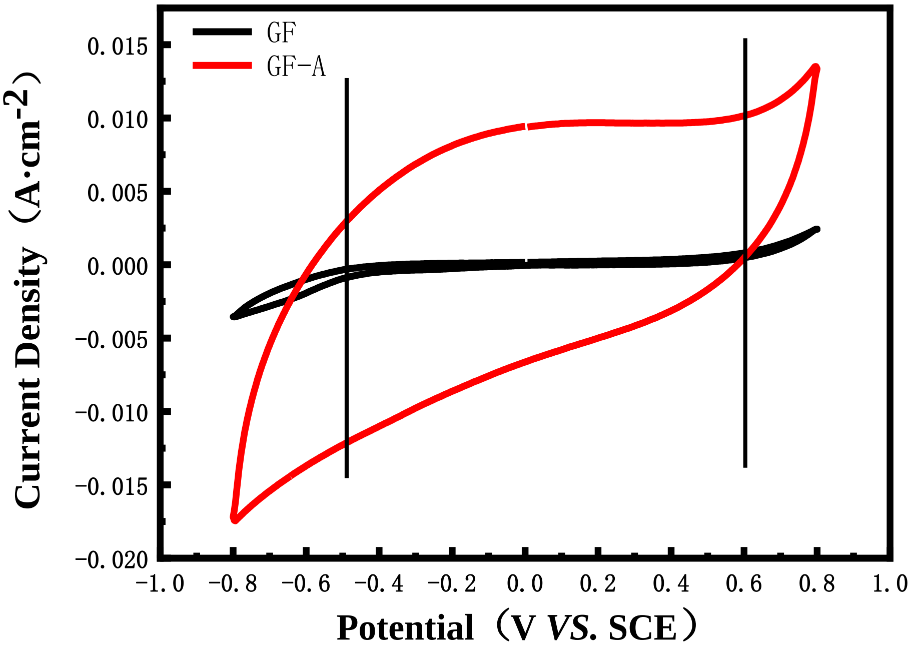

Figure S1 CV curve in 1 mol·L^{-1} NaCl electrolyte

Normally, the area where the electrode contacts the solution is the real area of the electrode, and the real area of the plate electrode is easily determined and measured. However, for the flow battery, the electrolyte is in a flowing state, and the reaction occurs only when the active material flows through the electrode. This requires designing a porous electrode, and it is necessary to smoothly pass the electrolyte through the electrode while maximizing the contact area of the electrode with the active material. The graphite felt electrode can well solve the above problems. However, since the graphite felt electrode is an electrode material formed by criss-crossing carbon fibers, a large amount of pores are formed in the space, so that the contact area is geometrically multiplied while the solution is in full contact with the electrode. In electrochemical experiments, increasing the effective reaction area can speed up the reaction rate and reduce electrochemical polarization.

Figure S1 shows the CV curves of the GF electrode and the GF-A electrode in a 1 mol•L^{-1} NaCl solution. The area of the GF electrode obtained by integrating x=-0.5 and

x=0.6 is 1.31×10^{-4}. The area selected from the GF-A electrode is 6.93×10^{-3}, indicating that the GF-A electrode capacitance is 53 times that of the GF electrode. The capacity is proportional to the area. Similarly, the true specific surface area of the GF-A electrode is 53 times that of the GF electrode. This may be due to the fact that the graphene-loaded catalyst greatly increases the effective reaction area and on the other hand improves the hydrophilicity.

XPS data

Table S2 GF and GF-A electrodes: various elemental contents and relative percentage

Samples	C_{1s}/at%	O_{1s}/at%	N_{1s}/at%	C/O	C/N	O/N
GF	92.1	7.9		11.65%		
GF-A	89.1	7.2	3.7	12.37%	24.08%	1.94%

Table S3 Experimental data from XPS spectra fitting based on the Shirley background

Samples	C 1s / %					O 1s / %		
	C-C	C-OH	C=O	-COOH	CO/CO_2	C=O	C-OH	O_2
GF	73.17	16.62	5.92	1.8	2.49	15	44	40
GF-A	72.32	18.79	5.1	2.13	1.67	31.35	27.48	41.17

Single-battery test section

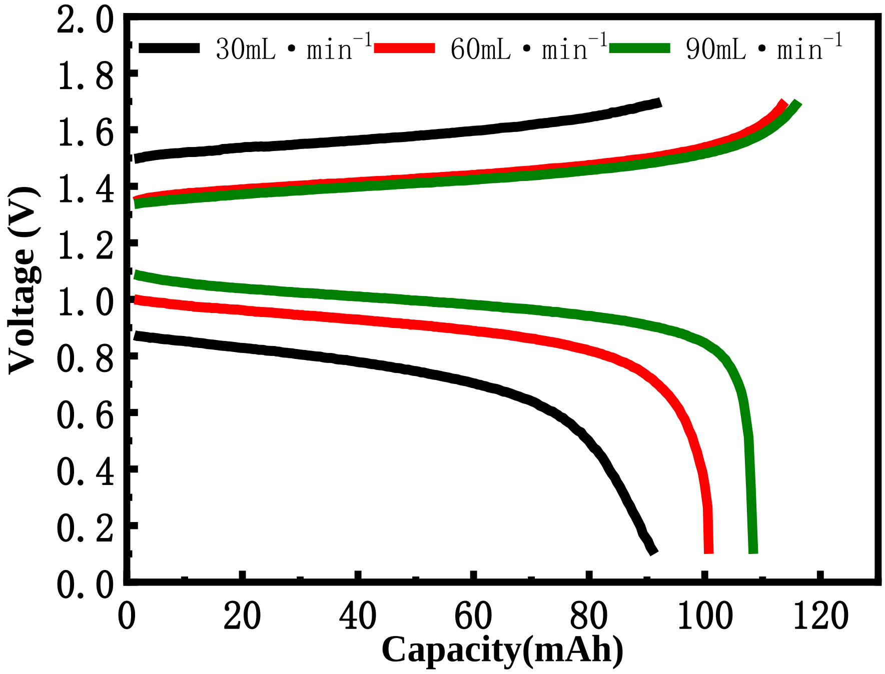

Figure S2 charge and discharge diagram of different flow rates of GF electrode

GF	CE	VE	EE	C%(C/C_{theory})	GF-A	CE	VE	EE	C%(C/C_{theory})
20	84.5	82.5	69.7	95.35	20	90.6	79.7	72.2	100
60	91.9	55.1	50.6	65.58	60	93.4	51.6	48.2	93.69

Table S4 the value of CE,VE,EE and C% for GF and GF-A electrode

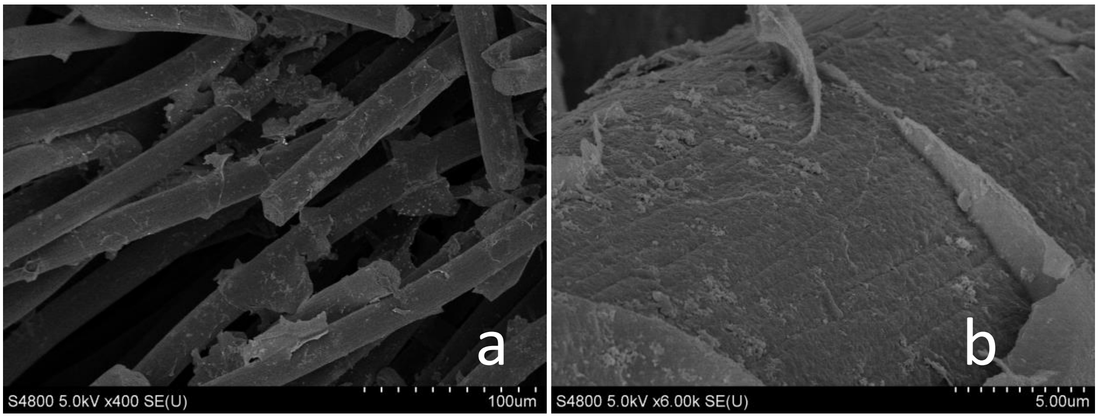

Figure S3 SEM images of: (a) GF-A, 400 times(b) GF-A, 6000 times
